# War Surgery in an Indian General Hospital in Mesopotamia

**Published:** 1917-12

**Authors:** Percival S. Connellan


The Bristol

Medico-Chirurgical Journal.

*" Scire est nescire, nisi id me
Scire alius sciret."*

DECEMBER, 1917.

### WAR SURGERY IN AN INDIAN GENERAL HOSPITAL IN MESOPOTAMIA.

BY

PERCIVAL S. CONNELLAN, I.M.S.

During the advance to Bagdad it fell to my lot to treat a considerable number of wounded in the Indian General Hospital to which I was attached. We were situated about half-way down the lines of communication, and to a large extent our work was that of a clearing hospital, combined with some of the advantages in equipment and staff of a general hospital.

The difficulties of transport in this country are well known. As far as the sick and wounded were concerned, it was done almost entirely by means of river steamers over a length of line 300 miles, which suddenly became extended to over 500 miles in the short space of about a fortnight.

The conveyance of wounded was arranged by stages of one or two days, followed by a night's rest, until they reached

Amarah, where for the first time we were able to give them a bath and put them in some comfort into beds.

Little can be done on a river transport ship beyond feeding and changing of the dressings of the more serious cases. Much good work was done in the Field Ambulances and Clearing Hospitals up the line, where every wound was dressed and urgent operations were performed.

Our equipment, as I have said, was that of a general hospital in tents, with an improvised operating theatre in a hut containing good theatre furniture, including two operating tables and a high-pressure steriliser.

A first-class X-ray outfit was invaluable.

The staff was small compared to that of a British hospital, my section of 200 beds having two medical officers, two sub-assistant surgeons (that valuable class of Indians trained in medicine and surgery, and without whom it is next to impossible to treat our Indian patients), eight ward orderlies (being sepoys who have had some hospital training), a couple of dressers and some menial personnel, and finally one nursing sister, to whose care and devotion some of my patients owe their lives, and all owe a debt of gratitude for comfort.

The character of the Indian sepoy patient differs widely from the British "Tommy," though their anatomy and physiology are the same.

One has to bear in mind that they are practically ignorant of the ways of western medicine, and broadly speaking they have little faith in it, but they are capable of a great deal of faith in the individual who is administering that treatment, and I have found it well to do nothing to discourage that faith, which maxim might be said to hold good in one's attitude toward one's patient of whatever creed or nationality.

Then, too, they are impatient to get home, and have but one guide to progress, the presence or absence of pain, leaving their shattered limbs to the care of the doctor so long as they remain painless, but often removing splints and dressings regardless of the consequence if uncomfortable, very much as a dumb animal would. Their power of endurance is great, but of mental resistance to disease there is very little.

*General lines of treatment.*—The wounded arrived in batches, and as a general rule I found them tired from the discomforts of their long journey, and suffering from a condition of mental exhaustion that made them very irritable; this I made it my first duty to treat.

The patient was at once put into bed with a complete change into hospital clothing. I think one has to be wounded oneself to realise what this means. To be incapacitated from looking after oneself by a wound, and then to lie in the blood and mud-soaked clothes that one has worn without change for days or even weeks, dependent on overworked orderlies for one's food, and to be carried from place to place, each move causing pain in the wound, is an experience calculated to exhaust the hardiest of us.

The next essential point I found was food, so each man was given a meal of whatever suited his condition best; for the more serious cases milk was the most asked for, for milk is to the Indian almost a stimulant and certainly a tonic. Hot soups or ordinary meals of meat, bread and vegetables for others.

When the patients were comfortably fed and rested in their beds, but not before, I have made it a practice to change every dressing; but I try to do this with as little disturbance to the patient as possible, and only on rare occasions have I found it necessary or advisable to change the form of splinting or give the patient an anæsthetic on the day of his arrival in hospital; for I think that more harm than good is done by disturbing his first night's sleep in a comfortable bed by the nauseating taste of chloroform or the change in position of a somewhat stiffened or cramped limb.

Morphia and soporifics I give unsparingly on the first night. By the next morning the patient's outlook on life is considerably improved, and he looks on his doctor with more confidence when he finds that he does not think of his wound alone, and he is more ready to bear the pain involved in the more thorough cleaning of his wound, or submit to being carried to the operating theatre, which to the sepoy on his first visit is more like a chamber of horrors than a place for the practice of the healing art.

In dealing with wounds my practice has been to interfere with nature as little as is consistent with ordinary laws of surgery, and a due regard to the ultimate condition and usefulness of the wounded part.

I regard the general resistance of the patient to sepsis as one of the most important factors in the healing of these wounds, and in this respect it has been observed that the sepoy is more resistant to sepsis than the British soldiers, perhaps owing to years of untreated cuts and bruises in his work at home in the fields giving him a certain power of immunity in the same way as he undoubtedly has a higher immunity to water-born and fly-carried diseases.

I would include the mental condition as an important part of the general resistance to disease, and to this end I try to keep the mind of my patient as healthily occupied and as free from worry as it is possible in a country about which it has been said that " the devil owned Mesopotamia and hell, and he elected to live in hell and leave Mesopotamia for us." Good food and plenty of extras such as milk, chickens, tinned fruits, eggs and vegetables, and a varied diet I regard as important, with the addition of alcohol, sweets and cigarettes if the patient desires them.

As regards local treatment, my aim has been to produce a useful result with sound healing as rapidly as possible, rather than a perhaps more perfect aim of shapeliness which would take longer, and might ultimately be of less use to a man who literally earns the bread he eats by his physical labour. This applies more particularly to fractures, which I will refer to again.

*Special local conditions.*—There are certain local conditions which make war in this country differ from that in most others. The long single line of communication up one of the most difficult of navigable rivers in the world for 500 miles makes it essential that there should be no congestion in any particular place, so that a steady and rapid evacuation of wounded may continue; and the length of time taken necessitates active medical and surgical treatment the whole time, whether the patient is stationary or travelling. One has to remember, too, that one's patient will continue to be treated by people under less favourable circumstances than that of a large hospital until he finally comes to rest in one of the hospitals in India, not as is the case in France in a few days, but in a few weeks.

One has also to be ready to do a sudden rapid expansion should there be a block anywhere in the communication. Sometimes I had to evacuate very rapidly to meet the requirements of the river transport ships, and at others I had to retain my patients longer than was necessary for their welfare for the same reason, though speaking generally I am left with an admiration for the way the transport has been managed, and the men who have managed it.

The climate of Mesopotamia can only be described as beastly for eight months of the year, good for two months, and wet and cold for two months; it is never a climate to which anyone would come for the benefit of his health, and finally it is not the climate that any of the troops fighting in it are accustomed to.

Water is very important to the Indian, so much so that he always describes a place as being good or bad by its water supply. When we talk of taking "a change of air," he speaks of a "change of water." The water of Mesopotamia is bad.

The character of the wounds differed somewhat from those that have been described in France, and from those that I have had to treat in the Dardanelles.

We are fighting here for a considerable extent on virgin soil, and the risk of tetanus is less, though it was not neglected, and most cases received a dose of antitetanic serum. I had only one case, and I heard of few others, even among the wounded Turks. During the early days of the advance, when it was almost entirely trench fighting, there was of course a large number of bomb wounds and of those due to close-range rifle bullets, and only a few due to high explosive shells.

Later there was a fair proportion of shrapnel wounds, and then finally, when the Turks had been dislodged, the majority of the wounds were long-range rifle bullet wounds, with in the majority of cases an absence of great severity.

All wounds were of course potentially septic, but the vast majority of clean perforations healed rapidly and well under any form of wet dressing, without any tendency to abscess formation if they were dressed frequently enough, even when the bone had been injured in transit.

The larger wounds were almost invariably septic as usual, but in the majority of cases nature responded well and required little assistance from the surgeon's knife.

*Treatment of wounds.*—Lotions. There has been a great deal of discussion about the various forms of lotions, pastes and powders used in the treatment of gun-shot wounds, and

I suppose everybody has his pet lotion, as every lotion has its advocate. In the treatment of these cases I have used mercury, carbolic, potassium permanganate and eusol lotions, hydrogen peroxide, salt solutions of various strength, and salt packs, iodine, bismuth paste, and various powders. I find that for ordinary use perchloride of mercury lotion of a strength of 1 in 2,000 is efficient, easily handled, retains its strength, is unaffected by climate, and can be easily prepared and obtained; for these reasons I have come back to using it as my standard lotion, after straying into the fields of the more complicated and newer lotions.

The same things may be said for the carbolic lotion, and it has the additional advantage of not spoiling the instruments; but I think it is a little more irritating, or rather that there are certain people susceptible to it, and I am one of them.

For large and septic wounds I have found nothing to equal permanganate of potash, and I have found its action to be more lasting than hydrogen peroxide, with which I have been rather disappointed; the latter I found useful in working its way into recesses and cleaning out a wound, but I think its action is mechanical, and there it stops. Permanganate of potash has the advantage of a colour index, which is, I think, more useful than knowing its strength, for the different waters which one mixes with it have a varying amount of material in them to be oxidised in these out-of-the-way places. I use it for irrigation. It is now difficult to obtain.

Eusol lotion disappointed me, because I had heard so much of its high qualities and powers of arresting sepsis, but in my hands I am sorry to say I have found it no more efficient than mercury lotion, and a great deal more uncertain. I think I may have been unfortunate in the preparations I obtained, for I understand that it is made from bleaching powder, and that is itself a very unstable compound. In a climate like this it has to be freshly made every day, and varies in strength from hour to hour, and one's nose seems to be the only guide to its strength. If used too strong it is capable of severe blistering, and if too weak it is useless, and finally it has to be used cold.

I find that most people now use it in conjunction with hypertonic salt solution. When I was treating British wounded from the Dardanelles at Port Said in May, 1915, I found the most marked effect of salt solution was to produce severe pain even with a 5 per cent. solution, and I had to reduce the strength to 2½ per cent. Recently, while treating Indians, I have been using 10 per cent., which caused no pain at all, and that has led me to the use of salt packs, which are little bags of salt which I place in the wound and cover with a very wet dressing or continuous irrigation, and I have been pleased with the results in chronic indolent septic cases; they produce pain, however, and tend to cause a little sloughing where the salt remains in contact with the tissue.

Iodine I use for the preparation of the skin, made in solution with spirit, and I think the spirit is more important than the iodine. I have had very little experience of pastes beyond bismuth or bismuth and iodoform made up with vaseline. I have found it useful in cases of sinus with deep suppuration where I wished to avoid frequent dressings. I have never known it do any harm.

Scarlet red I have found of value in chronic ulcers. I have also used calomel for these, and believe it does good in these cases, apart from any syphilitic taint. I use it for Oriental sores.

With regard to the actual dressing of the wound, I have listened to many discussions on the merits of large dressings and small dressings, gauze packs and tube drains.

Fresh air is undoubtedly one of the best treatments for a wound, but dust is not. Given ideal conditions, I would prefer to treat wounds without any covering at all, but in tents and among dust storms it is imperative to have a covering of some sort, and with a limited number of bed-sheets and clothes, it is necessary to have sufficient covering to absorb or spread out the discharge. The best material for this purpose is gauze, outside which I put wool in sufficient quantity to guard the wound from any minor injury, for one knows by personal experience how frequently a sore place becomes knocked, and patients I find always ask for plenty of wool. It is true that it shuts out the air, but I think the advantages in comfort of a big pad of wool outweigh those of the thin pad.

I do not as a rule put waterproof sheeting over the gauze, for I find it tends to make the wound and skin around sodden.

Then there is the question of drainage of the wound, and there is no doubt in my mind that a rubber drainage tube placed in a properly dependent position will drain a wound; but a wall of granulation tissue forms rapidly round it, and then it ceases to be of much use; for the first two days after a wound has been opened up for deep-seated pus I think it is the most efficient form of drainage. There are two other great objections raised to drainage tubes, and they are the risk of erosion into an artery, and necrosis of bone where the tube comes in contact with it; it is therefore well to be on one's guard, and not to put a tube near an important artery. I have no personal experience of this accident, nor have I been told of a specific case, but it is a danger to be avoided. With regard to the second objection, if the tube is removed in a couple of days I have found no evidence of necrosis afterwards.

Gauze inside a wound has the advantage of keeping the wound open, but it does not drain; there is no such thing as a gauze drain. How often has one seen the pus welling up from a sinus or deep part of a wound after the removal of the so-called gauze drain. This is due to the pus clogging the meshes of the gauze in the first place, and sometimes to the drying of the discharge upon the surface of the wound; both these difficulties can be overcome by frequent dressings, that is to change the dressing as soon as the gauze has absorbed as much of the discharge as it will before it has become clogged.

If the outside dressing be kept moist, the discharge will find its way beside the gauze to some extent.

The main use of gauze I find is to carry one's lotion into the recesses of the wound and retain it there, and this it does satisfactorily. I therefore use my gauze very wet with antiseptic lotion and lay it lightly into every part of the wound that I can get at, and do not rely upon it for drainage at all.

If the wound is properly opened at the time of operation it usually drains itself without much further assistance, and if it requires no operation it requires little or no artificial drainage. I think one of the greatest advances in the treatment of the wounds in this war has been the frequent and early change of dressings, doing away with the time-honoured policy that the first field dressing was the best, and might be left on for days.

It has been advocated that large and profusely-suppurating wounds may be left undressed for several days. I have not had the courage to try it.

*Operative treatment.*—The main class of operative treatment I have found necessary has been the removal of foreign bodies. The sepoy has an almost superstitious dread of a bullet or other foreign substance remaining in his body, and even if it is causing him no pain and lies in some unimportant part, he is not happy until it is out. I have found while putting on first field dressings in the field that almost invariably his first question is, "Has the bullet passed out?" and if one can tell him that it has he is much relieved; if the bullet is retained he regards it as a calamity. Very few of them would think of returning to the firing-line with a bullet inside them, and for this reason I make it a rule if possible to remove all foreign bodies where the operation will not endanger life or limb.

In this connection X-rays are essential, and I have made it a practice to have every case that I can X-rayed, and to be present myself in cases with foreign bodies or fractures, for no report by another person can give you that mental picture of the bone in all positions which is so valuable for the treatment of fractures, nor can another person describe the position of a foreign body so well as one can see it for oneself.

The other main field of surgery has been sepsis and the putting up of fractures on the various forms of splints.

In the absence of urgent symptoms I prefer to wait for a few days to see what nature is doing for the arrest of sepsis, but if there is any chance of pocketing of pus, the earlier it is attacked the better. Having decided that operation for freer drainage is necessary, the difficult question as to how much should be done has to be decided ; and here I have found no golden rule to guide me, for the general resistance to sepsis varies so enormously in different individuals, that in one case the mere evacuation of the pus is sufficient to cause the wound to heal rapidly, while in an almost similar wound, do what one will, the sepsis seems to spread. I therefore take each case on its merits, and keep in mind the patient's general resistance.

On the whole, I am in favour of conservative measures rather than of making large incisions ; if the operative interference is not extensive enough, it is easy to do more, but one cannot undo the work of a too free use of the knife.

In this connection the risk of toxæmia from the absorption of toxins from freshly-divided tissues has to be borne in mind, and one may find that one has done harm by suddenly poisoning the patient's system with a large dose of toxins.

Another difficult question I find to answer is, How much of the broken fragments of bone ought one to remove? And here again I would rather remove too little than too much. I remove all fragments lying free in the cavity, and so long as I can leave enough for the bone to unite, I remove those loose pieces which although attached to living structure are interfering with free drainage, but I do not remove pieces because there is a question as to their ability to live.

Fortunately I have had occasion to do very few amputations, as here again with the Indian sepoy circumstances are different from the British soldiers; for whereas to a British soldier who may be a clerk in an office or who can live a partially sedentary life an artificial limb properly fitted is often better than a hopelessly-fixed joint or crooked limb, this is not the case with the sepoy, who in many cases lacks the intelligence to use an artificial limb properly, or who has, as many have, a great prejudice against them. I think, speaking generally, he would rather keep a limb of any sort than have an artificial one.

*Fractures.*—The fractures I have treated here have been the same as I suppose they are everywhere else, that is they have been of every variety, from the simple fracture to the compound comminuted one. Many of them, if not all, were septic or associated with sepsis in the tissues around.

The most noticeable feature about them has been the slight degree of displacement, and often the absence of any of the signs of a fracture, which was only discovered by the X-rays.

In the case of septic fractures, I have found the best form of treatment is rest and fixation by splints with a weight extension, and I have not found that much extension has been necessary, especially while active suppuration is in progress, but the gentle and steady pull of weight over a pulley relieves much pain and keeps the limb at rest. I had to evacuate most of my cases as soon as the suppuration had subsided, and so had not much opportunity of observing the union of the bone. Fractures with a septic wound I have treated as simple fractures by early massage and movement, that is massage from the second day, passive movements on the fourth day, and active movements on the seventh day. I had nobody among my personnel who had been trained in massage, but most Indians have a crude knowledge of it and understand the value of rubbing, so it was not difficult to pick out a few and teach them the art of gentle rubbing which is so soothing to the patient, and in my opinion so beneficial to the repair of the wounded limb. My aim has been to get a strong, useful and free limb as rapidly as possible, and for this aim early massage and movement is the best treatment I know.

Fractures of the thigh I treated in nearly every case on Hey Groves' thigh splint. I found the same splint valuable for cases of bad compound fracture of the leg, as it is so easy to get at the wound for dressing purposes. I found two side splints placed inside the wire frame along the leg useful for steadying the limb, and as an alternative I used the long straight one; by changing from one to the other, the thigh and knee are kept from becoming cramped. For those with only small wounds I used Macintyre's splint for the first few days, and then swung them in a Salter's cradle, and finally sent them on in a modified box splint or a Croft's plaster splint.

For fractures of the humerus I found nothing better than simply bandaging the arm to the body for nearly all cases; I tried various forms of splints, but nearly always had to abandon them. Wounds about the elbow I treated with Hey Groves's simple wire frame, reinforced with a couple of side splints if necessary, and I also used it for big wounds of the forearm.

Fractures of the forearm bones rarely required extension, and X-ray showed very slight displacement in the majority of cases ; an anterior right-angled splint with a straight back splint I found as good as any, in many cases simple straight anterior and posterior splints were enough to keep the bones in good position.

*Wounds of special parts.*—Penetrating wounds of the head were conspicuously few. In some cases of scalp wounds the wound had been excised and sewn up before they arrived; they healed by the first intention.

Face wounds on the whole did well, presumably owing to the vascularity of the part, for they were all badly infected from the nose or mouth to start with.

The only case of secondary hemorrhage which I had was in a wound which had penetrated the floor of the nose; the hemorrhage stopped almost at once on removing a small piece of necrosed bone.

Abdominal wounds I found very interesting. I was struck by the insidious formation of pus without any rise of temperature. The best guide is the pulse and the presence of even slight rigidity; some cases showed no other sign, though on opening the abdomen I found a large quantity of pus.

I think the best treatment in these cases is to evacuate the pus and not to do too much. I simply open the abdomen, pack off the area and gently open the abscess with my finger, and carefully swab out the pus without breaking any adhesions, using a tube and if necessary a gauze localiser. I do not irrigate until the third day, by which time protective adhesions have formed, and I use permanganate of potash lotion, 1 in 10,000. I make no attempt to find the bullet.

I have had two cases which came under my care. Within the first twenty-four hours I operated on both, and in each case found no injury to viscera, the operation wound healing before the gun-shot wound; and I am sure this is the right treatment, as no harm is done by looking inside, and if injury has occurred it is better to deal with it before pus has had time to form, if one can be reasonably sure of one's asepsis.

I saw only a few cases of aneurysm, and the chief point that struck me about them was that they were well protected.

Wounds of the chest did well; none of them were tapped and none of them formed an empyema; several of them had slight surgical emphysema.

Wounds of the knee-joint if left alone did well, and I discovered as others have done the danger of removing a foreign body too early. I think, speaking generally, no attempt should be made to remove a foreign body for at least six weeks. The joint should be kept at rest on a splint. If the knee-joint is full of pus, I am in favour of tapping it once, and this failing, at once opening the whole joint by an incision across the patella ligament. They are one of the most difficult classes of case to treat.

Gas gangrene was fortunately uncommon. The chief complication was broncho-pheumonia, which is very fatal among Indians.

I have never seen a case diagnosed as shell shock in an Indian, though I have seen a few become insane through the stress of war.

I should like to express my admiration for the R.A.M.C. officers, both temporary and special reserve, who have given up so much at home and come to help us in the Indian hospitals, and who have done much to help the sick Indian soldier. In spite of the difficulties of not knowing his language or customs, they have strengthened the reputation for kindness and honour which it is the privilege of the medical profession to maintain.

My special thanks are due to Captain C. Milne, R.A.M.C., S.R., of Bath, who has shared the work of the section with me for some months.

